# Reconciling VEGF With VPF: The Importance of Increased Vascular Permeability for Stroma Formation in Tumors, Healing Wounds, and Chronic Inflammation

**DOI:** 10.3389/fcell.2021.660609

**Published:** 2021-03-23

**Authors:** Harold F. Dvorak

**Affiliations:** Department of Pathology, Beth Israel Deaconess Medical Center, Harvard Medical School, Boston, MA, United States

**Keywords:** VPF/VEGF, vascular permeability, tumors, wound healing, chronic inflammation, delayed hypersensitivity, angiogenesis, stroma

## Abstract

It is widely believed that vascular endothelial growth factor (VEGF) induces angiogenesis by its direct mitogenic and motogenic actions on vascular endothelial cells. However, these activities are only detected when endothelial cells are cultured at very low (0.1%) serum concentrations and would not be expected to take place at the much higher serum levels found in angiogenic sites *in vivo*. This conundrum can be resolved by recalling VEGF’s original function, that of an extremely potent vascular permeability factor (VPF). *In vivo* VPF/VEGF increases microvascular permeability such that whole plasma leaks into the tissues where it undergoes clotting by tissue factor that is expressed on tumor and host connective tissue cells to deposit fibrin and generate serum. By providing tissue support and by reprogramming the gene expression patterns of cells locally, fibrin and serum can together account for the formation of vascular connective tissue stroma. In sum, by increasing vascular permeability, VPF/VEGF triggers the “wound healing response,” setting in motion a fundamental pathophysiological process that induces the mature stroma that is found not only in healing wounds but also in solid tumors and chronic inflammatory diseases. Once initiated by increased vascular permeability, this response may be difficult to impede, perhaps contributing to the limited success of anti-VEGF therapies in treating cancer.

## Introduction

Normal tissues are comprised of two compartments: *parenchyma* and *stroma*. For example, the *parenchyma* of normal skin is the epidermis, a multi-layered squamous epithelium; skin’s *stroma* is the dermis, a “connective tissue” comprised of blood vessels, lymphatics, fibroblasts and their products (primarily collagen but also including glycosaminoglycans and proteoglycans), small amounts of interstitial fluid (a plasma protein-*poor* transudate of blood plasma), and small numbers of inflammatory cells (mostly macrophages and lymphocytes). The dermal stroma supports the epidermal parenchyma in three ways: it provides *nutrition* (oxygen, sugars, amino acids, etc.) and *waste disposal* (carbon dioxide, metabolic products) via the blood vasculature and lymphatics, and provides a collagenous matrix for physical *support*.

Like normal tissues, tumors are comprised of a parenchyma (the tumor cells) and a stroma that is of host origin. *All* tumors require stroma for survival and growth. As in normal tissues, stroma is necessary for tumor cell nutrition, waste product disposal, and support. However, not all tumors need to generate new stroma. Liquid tumors such as leukemias have inbuilt host stroma; the blood in which they circulate serves as stroma, affording abundant oxygen and nutrients as well as an efficient means for clearing waste products via the lungs, kidneys and bile system. Therefore, leukemic cells require no additional stroma beyond the blood plasma in which they circulate.

At least initially, when very small, *solid* tumors can make use of the stroma provided by the normal tissues in which they arise; indeed, some primary tumors (e.g., some glioblastomas) continue to “live off the land,” growing in cuffs around pre-existing host blood vessels (Holash et al., 1999). Tumor metastases to lungs and liver may also make use of pre-existing host blood vessels to supply their needs (Bridgeman et al., 2017). However, most solid primary tumors and their metastases require new stroma (Dvorak, 2015b, 2019). This newly generated stroma includes the same components found in the dermis and other normal tissues, though the overall composition and arrangement may differ. The *amount* of new stroma generated varies widely among different solid tumors. In many desmoplastic carcinomas, stroma comprises the bulk of the tumor mass. However, the amount of stroma is not correlated with tumor malignancy and some highly malignant tumors have relatively small amounts of stroma. Nonetheless, all tumors have and require stroma.

A critical question then arises: how do solid tumors generate the new stroma that they need for survival and growth? Often this question has been reduced to one of new blood vessel formation, i.e., how do tumors induce “angiogenesis”? However, angiogenic blood vessels, though necessary and important, are but one component of tumor stroma, and often only a minor component as measured by mass.

## Before Proceeding, a Necessary Primer on Vascular Permeability

An understanding of vascular permeability is foundational to a comprehension of the pathogenesis of stroma formation as occurs in tumors, healing wounds and chronic inflammatory diseases. The endothelium lining microvessels provides the primary barrier to the passage of plasma across the vasculature. In normal tissues, such as the dermis, capillaries exhibit a *basal* level of permeability such that plasma water and small solutes (generally ≤40 kD) pass readily through the interface between neighboring endothelial cells (Feng et al., 2000; Nagy et al., 2008, 2012; Dvorak,2015a,b). However, this inter-endothelial cell pathway does not permit the easy passage of larger molecules such as plasma proteins (≥60 kD). Nonetheless, plasma proteins do cross the normal capillary endothelium in small amounts, but primarily, it is thought, by a different pathway, i.e., by way of vesicles (caveolae). Caveolae are 70 nm vesicles that open to and then bud off from the luminal capillary endothelial surface, traverse the endothelial cytoplasm, and discharge their plasma cargo into the tissues after fusing with the abluminal plasma membrane (Majno and Palade, 1961 #152; Majno et al., 1961 #153; Palade, 1988 #271). Vascular permeabilizing agents such as histamine greatly increase the efflux of large molecules; at least in humans and guinea pigs, they act primarily on *venular* (rather than *capillary*) endothelium to open two potential pathways, about whose relative importance there remains controversy (Feng et al., 1996, 1997, 1999, 2000; Majno and Joris, 2004; Nagy et al., 2008, 2012; Dvorak,2015a,b): (1) A *transcellular* pathway involving the vesiculo-vacuolar organelle (VVO), an organelle comprised of hundreds of interconnected vesicles and vacuoles that spans venular cytoplasm from lumen to albumen. The stomata connecting these vesicles and vacuoles with each other and with the luminal and abluminal plasma membranes are normally closed but open in response to vascular permeabilizing agents to form transcellular channels through which whole plasma can flow. (2) An *intercellular* pathway generated by the pulling apart of adjacent endothelial cells to create gaps which allow the passage of whole plasma. The permeabilizing stimulus and resulting increased vascular permeability may be transient (as in the case of a mosquito bite) or chronic (as in tumors, healing wounds, and chronic inflammation), but in all cases results in an outpouring of a plasma protein-*rich* fluid that is both qualitatively and quantitatively different from the plasma protein-*poor* fluid bathing normal tissues. Further, when the stimulus is chronic, the capillaries and venules undergo profound structural changes, differentiating into ultrathin, intrinsically hyperpermeable *mother vessels*, as well as other abnormal vessel types (Dvorak et al., 1996; Pettersson et al., 2000; Nagy et al., 2006, 2007, 2010).

## Delayed Hypersensitivity as a Paradigm for Pathological Stroma Formation

Initial insights into the mechanisms of tumor stroma generation came from a surprising source, a form of cellular immunity known as *delayed hypersensitivity* (DH) (Colvin et al., 1973; Dvorak et al., 1974; Colvin and Dvorak, 1975). DH epitomizes the response of the body to allergens (e.g., poison ivy), foreign proteins (e.g., the tuberculin reaction), infectious agents (e.g., bacteria, viruses), and allografts. DH therefore has a prominent role in many important diseases, including tuberculosis, various other infections, contact allergy, rheumatoid arthritis, psoriasis and organ transplantation. DH is antibody-*independent*, mediated by T lymphocytes and macrophages, and can be passively transferred with lymphocytes but not with antibodies. Of course, many immunogens induce both cellular and humoral immune responses.

In the early 1970s, we reinvestigated DH skin reactions to tuberculin and other immunogens (Colvin et al., 1973; Dvorak et al., 1974; Colvin and Dvorak, 1975). We discovered two surprising new features of these reactions: (1) Hyperpermeability of the dermal vasculature to plasma proteins and other circulating macromolecules, and (2) Abundant dermal deposits of cross-linked fibrin. These two findings were linked causally. The hyperpermeability of the dermal microvasculature led to the extravasation of a plasma protein-*rich* exudate that included fibrinogen and other clotting factors, in contrast to the plasma-*poor* transudate of normal tissues. Further, clotting took place extra vascularly, converting extravasated plasma to *serum* and extravasated fibrinogen to cross-linked *fibrin*. Deposited fibrin took the form of a water trapping gel^1^ that was responsible for the induration characteristic of these reactions (Dvorak et al.,1979a,b #71).

These new findings were both unexpected and puzzling. First, increased vascular permeability had not previously been associated with cellular immunity; instead, microvascular hyperpermeability was thought to be a property of certain antibody-mediated reactions in which mast cells and basophils degranulate to release histamine, a well-known vascular permeabilizing factor. However, antihistamines did not inhibit DH reactions. Second, what was the significance, if any, of the clotting of plasma that generated serum and deposited fibrin?

## Stroma Generation in Solid Tumors Follows the Pattern of DH

Our new findings in DH reactions prompted us to determine whether similar events took place when tumors were used as antigens. For these studies we transplanted Line 1 and Line 10 bile duct carcinomas subcutaneously into guinea pigs, i.e., the same animal species preferred for studies of DH^2^. Over the course of a week or two, both tumors became organized such that clumps of tumor cells became dispersed within a newly generated vascularized collagenous stroma (Dvorak et al., 1979a). Line 1 tumors had a more abundant stroma than that of Line 10 tumors, but the organizational pattern was the same and was reproducible upon multiple transplants. Both tumors provoked a lymphocyte response, similar to that found in DH reactions elicited by tuberculin and other immunogens. Further, the vasculature supplying both tumors was hyperpermeable to circulating high molecular weight dextrans and plasma proteins and deposits of cross-linked fibrin were found intermixed within the collagenous stroma (Dvorak et al.,1979a,b).

The combination of lymphocyte infiltrate, leaky blood vessels and fibrin deposits implied that these tumors had elicited a DH-type immune response. However, evaluation of these tumors at very early times after transplant demonstrated that increased vascular permeability and clotting occurred independent of immunity. Within 48 h after transplant, long before the possibility of an immune response, tumors were already arranged in a pattern similar to that found at later times except that clumps of malignant cells were separated from each other not by vascularized collagenous stroma but by fluid-filled spaces that included strands of fibrillar material. Immunohistochemistry identified these fibrils as fibrin or fibrin-related proteins and electron microscopy demonstrated the characteristic banding pattern of cross-linked fibrin. Further, when subjected to a solvent that extracted soluble elements (such as fibrinogen, non-cross-linked fibrin, fibrin degradation products) much of this fibrillar material remained behind, confirming its identity as cross-linked fibrin (Dvorak et al., 1984; Dvorak, 2015b, 2019).

In sum, within hours of transplant, tumors became organized into a pattern in which tumor cell clumps were separated from each other by a provisional stroma comprised of fibrin gel that over time was replaced by vascularized connective tissue. Thus, although these tumors did indeed induce a cellular immune response, the immune response could not account for the vascular hyperpermeability and fibrin deposition demonstrated at early times after transplant. Similar fibrin deposits were found in the stroma of many different cancers growing *in situ* in patients (Dvorak et al., 1981a; Harris et al., 1982), establishing the generality of these findings and negating the possibility that the trauma of tumor transplantation or some other artifact was responsible for fibrin deposition.

## Cancer and the Clotting System

An association between cancer and blood clotting was not a new concept in the 1970s. Vascular thrombosis is a fairly common cause of cancer death, and abnormal hemostasis has long been associated with cancer [reviewed in Carr et al. (1985), Dvorak and Rickles (2005)]. Armand Trousseau long ago recognized migratory thrombophlebitis (Trousseau’s sign) as a predictor of underlying malignancy, the thought being that tumors shed procoagulant activities into the blood where they induce intravascular clotting. Some leukemias also express procoagulant activities that can induce disseminated intravascular coagulation. Several tumor cell-associated procoagulant activities have been identified. Most important among these is tissue factor, a cell membrane associated lipoprotein that activates the extrinsic clotting pathway and is widely expressed by tumor cells, including Line 1 and Line 10 carcinomas and many other animal and human tumor cells (Van De Water et al., 1985). In addition, these and many other tumors shed microparticles (exosomes) that also express tissue factor, allowing clotting to be initiated at a distance away from the tumor cells themselves (Dvorak et al., 1981b).

What was new from our work was the finding that clotting took place *extra vascularly*, such that fibrin was deposited and serum generated *outside* rather *within* the blood vasculature. As in DH reactions, extravascular clotting was readily explained by the hyperpermeability of the tumor vasculature that allowed extravasated fibrinogen and other clotting proteins to come into contact with tumor cell- and exosome-associated tissue factor.

## Fibrin Deposition Is a Generalized Response to Vascular Hyperpermeability

It was possible that there was something special about the DH and tumor microenvironments that allowed clotting of extravasated plasma. Therefore, to test the linkage between increased vascular permeability and clotting, we injected histamine and other vascular permeabilizing agents into normal guinea pig skin or other tissues and harvested the injection sites 20 min later (Dvorak et al., 1985). Such injections caused only brief pulses of increased vascular permeability that nonetheless resulted in deposits of cross-linked fibrin. Thus, even brief pulses of increased vascular permeability in otherwise normal tissues are sufficient to induce extravascular clotting and fibrin deposition. Clotting in these normal tissues was associated with fibroblasts and other host connective tissue cells that express tissue factor. That normal dermal cells express tissue factor is not surprising from an evolutionary point of view. Connective tissue cells are not normally exposed to the clotting proteins present in blood; however, when so exposed following tissue injury, the tissue factor they express is available to provide a protective function, activating clotting, depositing fibrin, and so limiting the extent of bleeding.

## Vascular Permeability Factor (VPF), the Mediator of Tumor-Associated Vascular Hyperpermeability

Because the cause of clotting in solid tumors was attributable to the tissue factor expressed by tumor and host connective tissue cells, the important unresolved question was the cause of the vascular hyperpermeability that led to extravasation of fibrinogen and other plasma proteins. To address this question, we cultured tumor cells in serum-free medium and tested supernatants for the presence of a possible secreted vascular permeability-inducing “factor” (Dvorak et al., 1979b; Senger et al., 1983). We found that a wide variety of tumors secreted such an activity. Using the Miles assay^3^ to assess column fractions, we purified this vascular permeability factor (VPF) to homogeneity. VPF turned out to be an N-glycosylated dimeric protein that rapidly induced permeability to plasma proteins and other circulating macromolecules with a molar potency some 50,000 times that of histamine (Senger et al., 1986; Yeo et al., 1991). It did not degranulate mast cells to release vascular permeabilizing factors such as histamine, nor was its activity inhibited by anti-histamines; instead, it acted directly on vascular endothelium. An antibody raised to the N-terminal sequence of VPF blocked its vascular permeabilizing activity and inhibited accumulation of ascites fluid in tumors growing in the peritoneal cavity (Senger et al., 1983). Many animal and human cancer cells were found to express this same protein (Senger et al., 1986).

**FIGURE 1:**
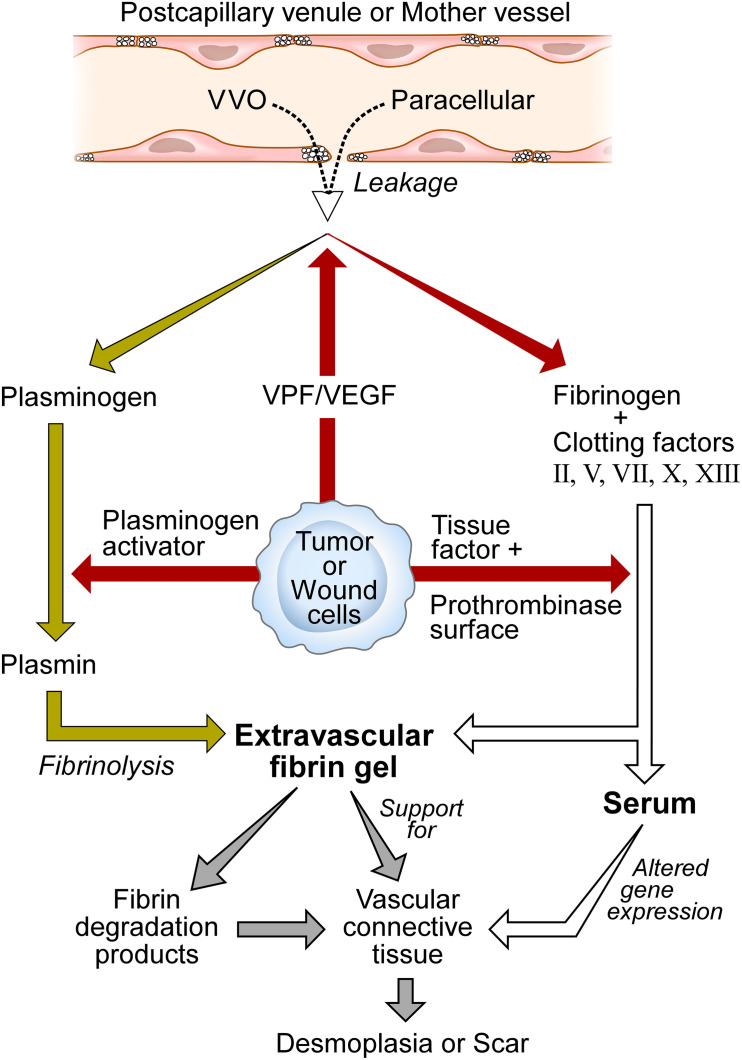
Schematic diagram of stroma formation in tumors and wounds. Vascular permeability factor/vascular endothelial growth factor (VPF/VEGF) initiates the wound healing response by increasing vascular permeability. As a result, whole plasma including plasma proteins extravasate by way of *transcellular* (VVO) and/or *paracellular* pathways. Tumor, connective tissue, and wound cells express tissue factor to trigger the extrinsic clotting system, generating serum, and depositing fibrin. Fibrin provides support for tumor cells and the ingrowth of new blood vessels and fibroblasts that synthesize collagen and other structural proteins. Fibrin degradation products are proangiogenic. Serum reprograms the gene expression patterns of tumor and host connective tissue cells. Over time, vascular connective tissue is replaced by dense fibrous connective tissue stroma termed scar in wounds and desmoplasia in tumors. Modified from Dvorak (2019, Figure 3) and Dvorak (b, Figure 1) with permissions.

Vascular permeability factor was thus the missing factor responsible for the vascular hyperpermeability characteristic of solid tumors. VPF rendered the microvasculature leaky to plasma and plasma proteins, resulting in extravascular clotting and fibrin deposition as tumor- and host cell-associated tissue factor triggered the extrinsic clotting system (Figure 1). Fibrin accumulation in tumors was limited by fibrinolysis that was mediated by another tumor cell-secreted product, plasminogen activator [reviewed in Dvorak (2015b, 2019)]. Plasminogen, like other plasma proteins, extravasates from leaky blood vessels, and is cleaved by plasminogen activator to generate the potent fibrinolytic protease plasmin. Thus, fibrin accumulation in tumors, as well as in other sites of increased vascular permeability, reflects a balance struck between clotting with fibrin deposition on the one hand and fibrinolysis on the other. The net amount of fibrin present initially seems to predict the amount of mature stroma that subsequently replaces it, perhaps thereby explaining differences in the amounts of mature stroma found among different solid tumors.

## Parallels Between Tumor Stroma Generation and Wound Healing

Speculations concerning a relationship between tumor stroma generation and wound healing go back at least as far as Virchow but were supported by little hard data. Nonetheless, two reports stand out. The first of these demonstrated that when the Rous sarcoma virus was injected intravenously into chickens, tumors formed preferentially at sites of incidental minor injury, i.e., at local sites where wound healing was taking place (Dolberg et al., 1985). A second important report by Majno demonstrated that in rat skin wounds there were two waves of increased vascular permeability [summarized in Majno and Joris (2004)]. The first wave was attributable to the injury itself which caused bleeding; this was generally staunched within a few minutes by platelet activation and clotting. However, a second wave of vascular hyperpermeability developed sometime later. This second phase of vascular hyperpermeability led to extravasation of whole plasma but not blood cells. It peaked at around 15 h after injury and persisted for a day or more. The mechanisms responsible for this second, unexpected round of vascular hyperpermeability were not known.

We confirmed Majno’s findings and demonstrated that VPF was responsible for this second wave of vascular hyperpermeability (Brown et al., 1992). Several hours after wounding, VPF expression was highly upregulated in regenerating epidermal cells immediately adjacent to wound sites, as well as in macrophages scattered in the dermis (Brown et al., 1992). Upregulation of VPF expression was attributable to the local hypoxia characteristic of wound sites that stimulates VPF expression through the HIF-1 pathway (Semenza, 2014). As a consequence of this heightened vascular permeability, fibrinogen and other plasma proteins leaked and clotting took place, converting extravasated plasma to serum and fibrinogen to cross-linked fibrin, just as occurred in tumors and in response to vascular permeabilizing agents in normal tissues. Further, as wound healing progressed, new blood vessels formed, relieving local hypoxia and thereby turning down VPF expression. A similar increase in VPF expression was found at other sites of wounding, e.g., myocardial infarcts in the heart (Li et al., 1996) and following local brain injury (Stiver et al., 2004). We concluded that tumors are “wounds that do not heal,” or, perhaps put better, tumors are wounds that continually exhibit elements of healing but do not heal the host (Dvorak, 1986, 2015b, 2019); instead, the healing process actually facilitates tumor survival and growth.

We subsequently demonstrated that VPF was also the previously unknown agent responsible for inducing the vascular hyperpermeability found in DH reactions (Brown et al., 1995) and in chronic inflammatory diseases such as psoriasis (Detmar et al., 1994) and rheumatoid arthritis (Fava et al., 1994). The same sequence of events followed in all of these examples: VPF induced vascular hyperpermeability which led to plasma extravasation, extravascular clotting, conversion of plasma to serum and extravasated fibrinogen to cross-linked fibrin. Fibrin was then degraded to varying extents by plasmin as extravasated plasma plasminogen was activated to plasmin by locally secreted plasminogen activator (Figure 1).

## Generation of Mature Tumor Stroma

Together the findings just summarized demonstrate the mechanisms by which increased vascular permeability leads to fibrin deposition in solid tumors, healing wounds, DH reactions, chronic inflammatory diseases, and in normal tissues in which vascular permeability to plasma and plasma proteins has been increased by VPF, histamine or other vascular permeabilizing agents. However, they do not explain the means by which tumor fibrin deposits are replaced over time by mature, vascularized connective tissue stroma. Therefore, there was considerable excitement when it was found that VPF was a weak though highly selective growth and pro-migratory factor for cultured vascular endothelium and that it was also able to induce angiogenesis *in vivo* (Connolly et al., 1989; Keck et al., 1989; Leung et al., 1989). VPF was immediately recognized as Judah Folkman’s long sought after “tumor angiogenesis factor” (Folkman, 1971) and was rechristened vascular endothelial growth factor (VEGF) (Leung et al., 1989). It was surmised that VPF/VEGF induced mature tumor stroma by causing endothelial cell division and migration.

However, there are two problems with this analysis. First, as noted above, angiogenic blood vessels comprise only one element of tumor stroma. In addition to new angiogenic blood vessels, solid tumors, and healing wounds feature greatly enlarged feeder arteries, draining veins, and several other abnormal vessel types (Pettersson et al., 2000; Fu et al., 2007; Nagy et al., 2007, 2010). How do these other vessel types form? Also not accounted for is the generation of important structural components such as collagen, proteoglycans, and glycosaminoglycans, as well as the fibroblasts and other cell types responsible for their synthesis; together these structural elements often comprise the bulk of the tumor mass.

The second and equally significant problem with this analysis is that of the highly restrictive conditions under which VPF/VEGF can act as an endothelial cell growth and motility factor. While VPF/VEGF is selectively mitogenic and motogenic for endothelial cells *in vitro*, such activity is detected only when endothelial cells are cultured in very low (typically 0.1%) serum. However, as shown above, the tumor microenvironment *in vivo* includes high serum concentrations that can approach those found when whole plasma is clotted. Tumor-induced ascites fluid may be taken as a proxy for the interstitial fluid in which solid tumors are bathed. Tumor ascites fluid typically has a high protein content, e.g., >25 g/dL (Tarn and Lapworth, 2010). While much of this protein content results from the death of tumor and other suspended cells, plasma-derived albumin content typically exceeds 2 g/dL, i.e., a plasma albumin content corresponding to 40% of that of whole plasma. Further, ascites fluid commonly becomes bloody and under these conditions plasma protein levels can approach those of whole blood. In such high serum concentrations, VPF/VEGF would not be expected to have mitogenic or motogenic activity on endothelial cells.

Together these two problems pose a conundrum. VPF/VEGF is able to induce the formation of mature, vascularized connective tissue stroma *in vivo* but apparently has no mitogenic or motogenic activity on endothelial cells under the conditions found in tumor stroma and of course in healing wounds. How is this conundrum to be resolved? It is possible that as yet unexplained circumstances *in vivo* allow VPF/VEGF to induce endothelial cell division and migration despite high local serum concentrations. However, there is a much simpler and I think more satisfactory explanation that depends on VPF/VEGF’s original activity as a highly potent vascular permeabilizing factor. By inducing vascular permeability *in vivo*, VPF/VEGF generates both *serum* and *fibrin* and together these can explain the formation of mature vascularized connective tissue stroma.

First, *serum* exerts powerful effects on cell gene expression. Chang et al. (2004) found that when fibroblasts were cultured in 10% serum, dramatic changes took place in the expression (up- or down-regulation) of a core group of some 677 genes, as compared when these cells were grown in low (0.1%) serum, i.e., under the conditions in which VPF/VEGF is effective as an endothelial cell mitogen and motogen^4^. Further, these same changes in gene expression are evident in important autochthonous human cancers (e.g., breast, prostate) where they correlate with the degree of tumor malignancy. The consequences of these multiple changes in gene expression are likely complex and difficult to assess, but the genes involved encode transcription factors, cell surface receptors, G protein signaling proteins, etc. and thus modulate many important cell pathways.

Second and equally important, *fibrin* has significant biological activities and is an important link in the chain of events leading to mature stroma generation. These activities were demonstrated convincingly by a simple experiment in which purified fibrin, prepared *in vitro*, was implanted in the skin of normal guinea pigs (Dvorak et al., 1987). The fibrin so deposited was gradually replaced by vascularized collagenous stroma, just as occurred in tumors and in healing wounds. The mechanisms by which these changes take place are not well understood, but fibrin has a number of properties that likely contribute [reviewed in Dvorak (2019)]. Fibrin provides structure as a provisional stroma. It binds many different integrins and so provides a promiscuous matrix for attachment, support and migration of fibroblasts, tumor and endothelial cells. In addition, fibrin can induce growth factor expression and it binds to and thereby sequesters growth factors, preventing their degradation by tissue proteases. Finally, several of fibrin’s breakdown products have angiogenic activity.

In sum, once VPF/VEGF increases vascular permeability, clotting is activated in the extravascular space to generate serum and fibrin which together initiate a chain of events that leads to the formation of mature vascularized connective tissue stroma (Figure 1). The details of this process are likely to be complex and remain to be fully ascertained^5^. However, after VPF/VEGF has induced increased vascular permeability, there would seem to be no further need for it to act as an endothelial cell growth or motility factor.

## A New Hypothesis

The data summarized here have led to a new hypothesis that can better explain the mechanisms by which VPF/VEGF acts *in vivo* to induce stroma formation; it also offers a possible explanation for the limited effectiveness of anti-VEGF cancer therapies. The hypothesis states that VPF/VEGF induces stroma primarily by way of its potent “VPF” function. Increased vascular permeability introduces a plasma protein-rich exudate into tissues that sets in motion a fundamental pathophysiological process, the “wound healing response,” which, once triggered, proceeds on “autopilot” (Figure 1). Tissue factor, expressed by many tumor cells and also by host connective tissue cells, initiates clotting of extravasated plasma to generate serum and to deposit fibrin; serum dramatically alters the gene expression pattern of tissue cells and, together with fibrin, promotes and supports the ingrowth of fibroblasts and new blood vessels. Because tumors and healing wounds are bathed in protein-rich serum, VEGF’s mitogenic and motogenic actions on endothelial cells are likely to be severely limited. Over time fibrin is replaced by vascular connective tissue stroma, and finally by largely avascular connective tissue termed *scar* in wounds and *desmoplasia* in tumors. Simply introducing purified fibrin prepared *in vitro* into tissues can replicate this response (Dvorak et al., 1987 #75). Tumors thus can be regarded as parasites that by secreting VPF/VEGF trick the host into thinking that a wound is present, thereby inducing the host to trigger the wound healing response. In this manner, tumors induce the stroma they require for survival and growth. By continuing to secrete VPF/VEGF indefinitely, tumors behave as wounds that are always healing but never heal. The healing process benefits the tumor at the expense of the host. Chronic inflammatory diseases make use of the same wound healing process, again by secreting VPF/VEGF. Anti-VEGF approaches to cancer have met with limited success, perhaps because once plasma has leaked into tissues, a powerful process has been set in motion that is difficult to impede.

## Data Availability Statement

The original contributions presented in the study are included in the article/supplementary material, further inquiries can be directed to the corresponding author.

## Ethics Statement

The animal study was reviewed and approved by Beth Israel Deaconess Animal Care Committee.

## Author Contributions

HFD was entirely responsible for the work reported here.

## Conflict of Interest

The author declares that the research was conducted in the absence of any commercial or financial relationships that could be construed as a potential conflict of interest.

## Footnotes

1 Gels are semi-solid structures in which small amounts of polymers of various types (in this case cross-linked fibrin) trap large amounts of fluid (here plasma), thus restricting fluid flow. Common examples include gelatin (“Jello”), and, of course, blood clots that form within blood vessels *in vivo* or in blood or plasma that is allowed to clot in a test tube.

2 Guinea pigs provide a better animal model of human cellular immunity (and probably of cancer) than mice. However, guinea pigs suffer from a number of disadvantages that have limited their usefulness: they are more expensive to purchase and maintain, more difficult to inbreed (only two inbred strains are currently available), have few available transplantable tumor strains, and their genetics have been much less well studied.

3 Ashley Miles developed an assay for evaluating the permeability inducing properties of small molecules such as histamine (Feldberg and Miles, 1953). We used this assay to assess the VPF produced by tumor cells (Dvorak et al., 1979b; Senger et al., 1983). Putative permeability inducing agents were injected intradermally into the depilated flank skin of guinea pigs. Evans blue dye was then injected intravenously. This low molecular weight dye binds to plasma proteins and so tracks their distribution, generating prominent blue spots in skin sites injected with factors that induce vascular permeability to macromolecules. Permeability can be quantitated by measuring the amount of accumulated dye (Sitohy et al., 2017).

4 It would be desirable to repeat these experiments with endothelial cells, but that is difficult because endothelial cells do not do well when cultured for long in low serum concentrations.

5 For example, other leaked plasma proteins such as fibronectin, vitronectin, etc. have known biological activities that are likely to contribute but their roles in stroma formation have not as yet been investigated.
